# Vector Competence of the Tick *Ixodes ricinus* for Transmission of *Bartonella birtlesii*

**DOI:** 10.1371/journal.pntd.0001186

**Published:** 2011-05-31

**Authors:** Caroline Reis, Martine Cote, Danielle Le Rhun, Benoit Lecuelle, Michael L. Levin, Muriel Vayssier-Taussat, Sarah I. Bonnet

**Affiliations:** 1 Institut National de la Recherche Agronomique, USC INRA Bartonella-Tiques, ANSES, Maisons-Alfort, France; 2 École Nationale Vétérinaire d'Alfort, Centre de Recherche Biomedicale, Maisons-Alfort, France; 3 Medical Entomology Laboratory, Centers for Disease Control and Prevention, Atlanta, Georgia, United States of America; University of Texas Medical Branch, United States of America

## Abstract

*Bartonella* spp. are facultative intracellular vector-borne bacteria associated with several emerging diseases in humans and animals all over the world. The potential for involvement of ticks in transmission of *Bartonella* spp. has been heartily debated for many years. However, most of the data supporting bartonellae transmission by ticks come from molecular and serological epidemiological surveys in humans and animals providing only indirect evidences without a direct proof of tick vector competence for transmission of bartonellae. We used a murine model to assess the vector competence of *Ixodes ricinus* for *Bartonella birtlesii*. Larval and nymphal *I. ricinus* were fed on a *B. birtlesii*-infected mouse. The nymphs successfully transmitted *B. birtlesii* to naïve mice as bacteria were recovered from both the mouse blood and liver at seven and 16 days after tick bites. The female adults successfully emitted the bacteria into uninfected blood after three or more days of tick attachment, when fed via membrane feeding system. Histochemical staining showed the presence of bacteria in salivary glands and muscle tissues of partially engorged adult ticks, which had molted from the infected nymphs. These results confirm the vector competence of *I. ricinus* for *B. birtlesii* and represent the first *in vivo* demonstration of a *Bartonella* sp. transmission by ticks. Consequently, bartonelloses should be now included in the differential diagnosis for patients exposed to tick bites.

## Author Summary

*Bartonella* spp. are bacteria that infect the red blood cells and that are associated with several diseases in humans and animals all over the world. They are transmitted by arthropod vectors including fleas, lice and sand-flies, but new potential vectors are suspected and in particular ticks. Diseases transmitted by ticks, currently in emergence, have diverse etiology (viral, bacterial, parasitic) and are responsible for high morbidity and mortality rates around the world. The potential for involvement of ticks in transmission of *Bartonella* spp. has been heartily debated for many years because of the numerous but indirect proofs of its existence. In this study, the authors used a murine model to assess the ability of the tick *Ixodes ricinus* to transmit *Bartonella* bacteria to mice. Results of the study confirm the vector competence of *I. ricinus* and represent the first *in vivo* demonstration of a *Bartonella* sp. transmission by ticks. Consequently, bartonelloses should be now included in the differential diagnosis for patients exposed to tick bites.

## Introduction

*Bartonella* spp. are facultative intracellular gram-negative bacteria, which commonly infect mammals, particularly rodents. Some of these are associated with emerging or re-emerging diseases in humans and animals [1]. To date, 13 *Bartonella* species or subspecies have been associated with a large spectrum of clinical syndromes in humans including Carrion's disease, trench fever, cat scratch disease, bacillary angiomatosis, Parinaud's oculoglandular syndrome, endocarditis, peliosis hepatis, myocarditis, neuroretinitis, fever, fatigue and neurological symptoms [2]. Although all bartonellae are presumed to be transmitted by arthropods, primary vectors have been identified with certainty for only five *Bartonella* spp.: the louse *Pediculus humanus humanus* transmits *B. quintana* [3], the cat flea *Ctenocephalides felis* is responsible for the transmission of *B. henselae* [4], the sand fly *Lutzomyia verrucarum* is the vector of *B. bacilliformis* [5], and the flea *Ctenophthalmus nobilis* is implicated in the transmission of *B. grahamii* and *B. taylorii* to bank voles [6].

The potential for involvement of ticks in transmission of *Bartonella* spp. has been heartily debated for many years (see reviews by [7]–[9]). However, most of the data supporting bartonellae transmission by ticks come from molecular and serological epidemiological surveys in humans and animals providing only indirect evidences without a direct proof of tick vector competence for bartonellae.

The only direct evidence of transmission of a *Bartonella* sp. by ticks to a susceptible animal was reported in 1926 by Noguchi who described experimental transmission of *B. bacilliformis* by *Dermacentor andersoni* [10]. In that study, adult *D. andersoni* ticks, which had been fed for several days upon infected monkeys, were allowed to reattach to naïve animals. These recipient naïve monkeys became infected, likely because of mechanical transfer of the pathogen on blood-contaminated mouth parts. Neither the tick's vector competence nor bacterial transtadial transmission throughout the tick life's cycle were assessed.

A recent study using an artificial feeding system provided first experimental data supporting vector competence of ticks for bartonellae [11]. Immature *I. ricinus* ticks were able to acquire *B. henselae* while feeding on artificially infected blood, maintain the pathogen through the molt, and secreted it into uninfected blood during the subsequent artificial feeding. Cats inoculated with dissected salivary gland of these ticks developed typical *B. henselae* infection, proving the viability of transstadially passaged bacteria. However, ticks were fed via an artificial feeding system on blood supplemented with bacteria just prior the feeding that does not reflect natural infection of reservoir animals. Therefore, experimental transmission studies using infected ticks and live susceptible animals are required to unequivocally demonstrate the vector competence.

*B. birtlesii* sp. nov. was originally isolated from wild rodents (*Apodemus* spp.) [12] and later shown to be infectious for laboratory mice [13], [14]. Considering the high natural frequency of infestation in wild rodents with *I. ricinus*, we assessed vector competence of this tick species for *B. birtlesii* by demonstrating its ability to acquire the pathogen from an infected host and transmit it to naïve susceptible animals during the subsequent feeding.

## Materials and Methods

### Ethics Statement

This study was carried out in strict accordance with the good animal care practise of the recommendations of the European guidelines. The protocol was approved by the Committee on the Ethics of Animal Experiments of the national Veterinary School of Alfort (Permit Number: 2008-11). All efforts were made to minimize suffering of animals.

### Ticks

All experiments were performed with *Ixodes ricinus* colony reared in our laboratory at 21°C and 95% relative humidity, under a 12 h light/dark cycle. For ticks colony maintenance, nymph and adult ticks were fed on uninfected rabbits (HYPHARM, Roussay, France), while larvae were fed on sheep blood (bioMérieux, Lyon, France) using the artificial membrane feeding technique previously described [15]. At each developmental stage, ticks were starved for at least three months between molting and the next feeding.

### Bacterial strain

*Bartonella birtlesii* (IBS325^T^ strain [12]) was grown on 5% defibrinated sheep blood Columbia agar plates (CBA) incubated at 35°C with 5% CO_2_. After 5 days, bacteria were harvested and suspended in sterile phosphate-buffered saline (PBS) immediately before being used for mouse infection.

### Mouse antiserum against *B. birtlesii*

Specific immune serum was generated by subcutaneous injection immunization of a Balb/C mouse (Charles River Laboratories, L'Arbresle, France) with 10^8^ CFU of *B. birtlesii* after a freeze-thaw step, and with a boost two weeks later. Blood was collected 26 days after the boost from the retro-orbital sinus and the serum was stored at −20°C.

### Mouse infection with *B. birtlesii*

A 4-weeks old OF1 female mouse (Charles River Laboratories) was experimentally infected by intravenous injection in the tail vein with *B. birtlesii* (5×10^8^ CFU in 100 µl of PBS). Blood samples were collected from the retro orbital sinus at seven, thirteen and nineteen days post infection, and the presence of *Bartonella* DNA was confirmed by semi-nested PCR as previously described [11].

### Tick feeding on *B. birtlesii*-infected mouse

For tick infestation, the *B. birtlesii*-infected mouse was briefly anaesthetized with 3% Isoflorane and a plastic cap opened at the top was glued on its shaved back with wax as described [16]. On days 13 and 14 postinoculation, hungry larvae (approximately 150) and nymphs (25) were placed in the cap, which was sealed with sticking plaster. Ticks were allowed to feed on the mouse for five days. At that time, the cap was opened, and the engorged ticks were collected and stored under standard conditions described above for molting into the next stage.

### *B. birtlesii* transmission from nymphs to mice

Nymphs fed as larvae upon the *B. birtlesii*-infected mouse were placed on naïve uninfected mice at approximately 3 months after the molt in order to evaluate bacterial transmission from ticks to mice. Three 4-weeks old OF1 naïve female mice were each infested with 8 nymphs (24 ticks in total) as described above. Ticks were allowed to feed until repletion.

Blood samples were collected from each mouse on the day of infestation before tick attachment (day 0) and at seven and 16 days after tick attachment. Mouse blood (25 ul) was incubated in 500 ml of Schneider Drosophila medium for 6 days at 35°C, 5% CO_2_ as previously described [17]. As *B. birtlesii* does not grow on blood agar after liquid medium culture (unpublished data), the presence of bacteria was confirmed by 2 methods: semi-nested PCR of *Bartonella* spp. 16S DNA as previously described [11] and immunofluorecence assay on 100 µl of the cell suspension. Briefly, cytospin is used to spin cell suspension onto the slide, which were fixed with 4% paraformaldehyde and washed in PBS. Slides were covered with mouse anti- *B. birtlesii* serum diluted at 1∶150 in PBS and incubated for 45 min. After washing, slides were incubated for 20 min with an anti-mouse secondary antibody (Alexa Fluor® 488 goat anti–mouse IgG, Invitrogen) diluted per manufacturer's specifications. Samples were then mounted in VECTASHIELD® Fluorescent Mounting Media (Vector Laboratories, Peterborough, UK) and examined under microscope.

At Day 16, the mice were euthanized and the livers were removed. Half of the liver was stored at −80°C, the other part was homogenized in 500 µL of F12 medium (Invitrogen, Cergy Pontoise, France). 250 µL of the homogenate were spread on CBA plates incubated at 35°C with 5% CO_2_. The plates were checked daily for bacterial growth, and the identity of appearing bacterial colonies as *B. birtlesii* was confirmed by nested-PCR amplification of *Bartonella* spp 16S RNA encoding gene followed by sequencing of the 337-bp amplified fragment as previously described [11].

### Localization of *B. birtlesii* in adult ticks

Female *I. ricinus* derived from nymphs that fed upon the *B. birtlesii*-infected mouse were fed four months later by membrane feeding technique as previously described [11], [15]. Thirteen females from the infected cohort were placed on a membrane feeder together with 13 males from our uninfected colony (for mating) and fed on sheep blood (bioMérieux) changed every 24 h. After tick attachment, the presence of *B. birtlesii* DNA in the used blood was detected by semi-nested PCR as previously described [11]. Once *Bartonella* spp DNA had been detected in blood, four females were removed and used for immunohistological assay. Two females from an uninfected cohort feeding simultaneously on a separate feeder were used as control.

The partially engorged female ticks were fixed in their entirety, 15 min in Carnoy's solution (3∶1, absolute ethyl alcool∶glacial acetic acid) before cutting the legs, and then left over night in the same fixative. Ticks were washed twice in 70% ethanol for 15 minutes, once in 95% ethanol for 1 hour and 4 times in 100% ethanol for 1 hour. Finally ticks were washed 3 times in butanol for 24 h before embedding in paraffin. For immunohistochemistry analysis, 4-µm thick sections were cut, dewaxed and pretreated for 6 min. with protéinase K (Sigma) at 37°C and in 3% hydrogen peroxide (Gifrer, Decines, France) for 10 min. at room temperature. Sections were then blocked for 20 min with 20% normal goat serum (Dako, Glostrup, Denmark). Mouse antiserum against *B. birtlesii*, diluted at 1∶150 was used as primary antibodies and incubated on slides in 2% BSA (Sigma) for 1 h at 37°C. The corresponding pre-immune serum was used as negative control. Anti-mouse (Dako) biotinylated secondary antibodies were then incubated on slides in 2% BSA for 30 min and antigen-Antibody binding was revealed with streptavidin-PAL (Dako) and Fast-Red Substrate for immunoperoxidase (Dako), according to the manufacturer's instructions. The slides were counterstained with Gill's hematoxylin (Surgipath, Peterborough, UK) and examined under microscope with magnification ×400.

## Results

### Transmission of *B. birtlesii* to mice by nymphal *I. ricinus*

PCR amplification of *Bartonella* spp. DNA in blood samples collected from the mouse infected with *B. birtlesii* showed that the mouse was bacteremic at days seven, 13 and 19 postinoculation. Therefore, ticks were placed on this mouse at days 13 and 14. After repletion, a total of 120 engorged larvae and 25 engorged nymphs were allowed to molt to nymphal and adult stage, respectively.

In order to assess the ability of *I. ricinus* nymphs acquisition-fed as larvae upon an infected mouse to transmit *B. birtlesii* to a susceptible host, 24 of these nymphs were allowed to feed on three uninfected mice −8 per mouse. Of these, a total of 11 ticks fed to repletion – three, two and six from each of the mice.

**Figure 1:**
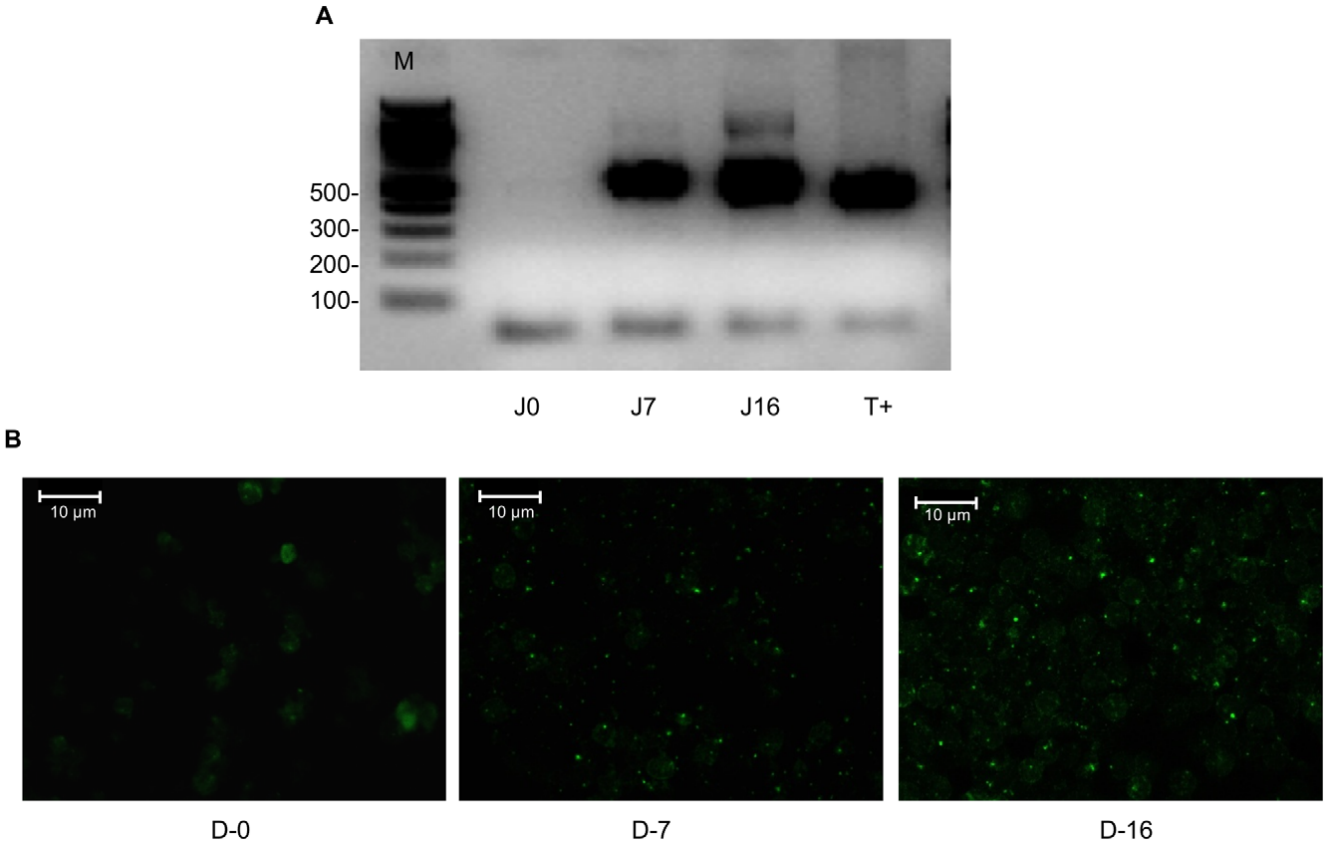
Mouse infection by nymph ticks. Detection of *Bartonella* in 6-day old liquid medium cultures inoculated with blood of a mouse fed upon by *B. birtlesii*-infected *I. ricinus* nymphs by A. semi-nested PCR. Lines D0, D7, and D16 represent blood samples taken on days 0, 7, and 16 after tick placement respectively; T+ – *B. birtlesii* DNA; M – molecular mass marker. B. immunofluorescence assay. D-0, D-7, and D-16 represent blood samples taken on days 0, 7, and 16 after tick placement respectively.

PCR detected the presence of *Bartonella* spp. DNA in Schneider Drosophila medium inoculated with blood samples from each of the three mice on days seven and 16, but not on day zero (Figure 1A). All amplified fragments were 100% identical to the *B. birtlesii* corresponding fragment of the 16S rRNA gene (accession number AF204274). *B. birtlesii* was also detected in the same samples by immunofluorescence (Figure 1B). This confirms the presence and viability of *B. birtlesii* bacteria in the blood of mice fed upon by *B. birtlesii*-infected ticks.

In addition, *B. birtlesii* colonies (also confirmed by PCR amplification and sequencing) were isolated from livers of the three recipient mice, demonstrating persistence of live bacteria for at least 16 days after mice had been bitten by infected nymphs. Identical results were obtained for all three recipient mice in both assays.

### *B. birtlesii* in adult *I. ricinus*

**Figure 2:**
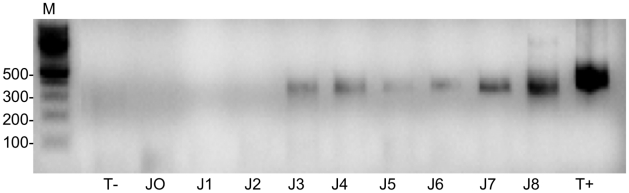
Blood contamination by female ticks. Detection of *Bartonella* DNA by semi-nested PCR in the blood from a feeder after placement of *B. birtlesii*-infected adult *I. ricinus* on the membrane: Lines D0–D8 represent blood samples taken on days 0 through 8 after tick placement; M – molecular mass marker.; T− and T+ – negative (distilled water) and positive controls (*B. birtlesii* DNA).

Thirteen female *I. ricinus* fed at the preceding nymphal life stage upon a *B. birtlesii*–infected mouse were re-fed with uninfected sheep blood on a membrane feeder. Blood samples were withdrawn from the feeder every 24 h during the 8–day feeding period to detect the presence of *B. birtlesii* DNA. *B. birtlesii* DNA was detected in samples drawn on days three through eight of tick attachment (Figure 2), indicating that adult ticks were successfully emitting the bacteria into the previously uninfected blood during feeding.

**Figure 3:**
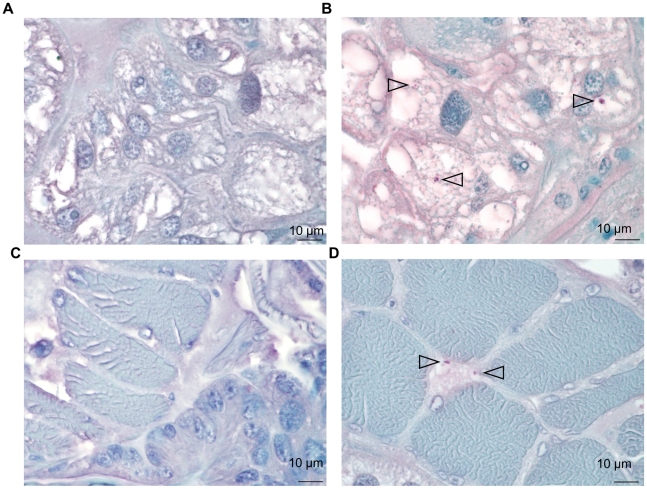
Ticks infection on mouse. Detection of *B. birtlesii* in adult *I. ricinus* salivary glands (A, B) and muscle tissues (C, D) sections colored with hemalun-eosin, by histochemical staining: A & C – uninfected ticks; B & D – infected ticks. Bacteria are indicated with arrows.

Four partially engorged females from the infected cohort and two partially engorged uninfected females were detached from the respective membrane feeders at 72 h post-attachment and used for histological examination. *B. birtlesii* bacilli were identified as dense particles of approximately 1 µm both in the cytoplasm of salivary gland cells and at the periphery of striated muscle section of all four ticks from the infected cohort, while no bacteria could be detected on uninfected ticks (Figure 3). No bacteria were detected in the midgut of the ticks (data not shown).

## Discussion

The question of whether any of the *Bartonella* spp. may possibly be transmitted by ticks has been debated for several years. Indeed, although it is believed that most *Bartonella* spp. are transmitted by an arthropod vector, these pathogens are always associated with erythrocytes and endothelium in their vertebrate hosts, and the ability of these bacteria to survive for many weeks and months between successive tick feedings in the absence of such cells is uncertain.

Numerous data have been published to date regarding identification of *Bartonella* DNA in both engorged ticks collected from their natural hosts and questing ticks collected from the environment (for detailed reviews see [7], [9]). As various *Bartonella* spp are common in wild and domestic animals, acquisition of these erythrocyte associated microorganisms by feeding ticks with a blood meal can be expected, and thus detection of bacterial DNA in engorged or partially engorged ticks does not add to the debate. However, positive PCR results in questing ticks do indicate that the bacterium (or at least its DNA) can survive in the tick through the molt from one life stage to another. In addition, a number of studies have reported co-infections in both humans and animals with *Bartonella* spp. and known tick-borne pathogens such as *Borrelia* spp., *Anaplasma* spp. or *Babesia* spp., suggesting that these might be co-transmitted by the same vectors [12]–[22]. *Bartonella* spp have also been detected by either PCR, serology, or culture in humans and animals after tick bites without any known contact with other arthropods [19], [23], [24], [25]. Recently, Angelakis et al. reported detection of *B. henselae* infection in three patients, who developed scalp eschar and neck lymphadenopathy following tick bites [26]. A *Dermacentor* sp. tick removed from one of these patients contained DNA of *B. henselae*, although it is unclear whether the person acquired an infection from the tick, or the tick from the person.

Our previous study demonstrated an innate ability of live *B. henselae* to be ingested by *I. ricinus* ticks with the blood-meal, maintained transstadially, and discharged again during the subsequent feeding [11]. In that study, however, ticks were acquisition-fed continuously on membrane feeders on blood containing 10^6^ CFU/ml. This concentration is the one that could be encountered in an infected cats, however, the experimental model remains an experimental model and does not reproduce ideally the natural conditions of pathogen transmission using ticks and animals and therefore, the vector competence of ticks could not be definitively established. The present study used live hosts as both the source and the recipients of bacterial infection in order to confirm vector competence of *I. ricinus* for a *Bartonella* sp.

Because of biosafety concerns associated with tick feeding upon cats infected with *B. henselae*, we decided to use a mouse model of *B. birtlesii* infection that has been studied in our laboratories for several years. The *B. birtlesii* strain used in this study was a low passage isolates from a field mouse *Apodemus* sp. [12]. Using this model, we showed that *I. ricinus* larvae and nymphs placed on an infected animal at the peak of bacteremia were able to acquire *B. birtlesii* from the host. Nymphs, infected at the larval stage, were able to inject *B. birtlesii* into mice, which in turn became bacteremic. Judging by the results of blood-PCR, the recipient mice developed bacteremia within seven days after placement of *Bartonella*-infected ticks and remained bacteremic at least until day 16. This timetable is comparable with those observed when mice were needle-inoculated with the same pathogen [13], [14], [27], [28]. Notably, we have re-isolated *B. birtlesii* from the liver of tick-infected mice, which confirms colonization of that organ by the pathogen observed earlier using needle-inoculation (unpublished data - MVT; [29]).

When we placed a cohort of infected adult ticks on a membrane feeder, *Bartonella* DNA was detected in all samples of the used blood removed later than 72 hours, but not in those tested at 24 and 48 hours. Similar results were obtained in our previous study [11]. Interpretation of these results requires several important considerations. Our previous experience shows that ticks placed on a membrane feeder may take up to 48 hours or even longer to find an attachment place, lacerate the skin-membrane, produce the cement cone, and to begin feeding. Once ticks are feeding on a membrane feeder, a few microliters of tick saliva are mixed with five ml of blood contained in the feeder resulting in a colossal dilution effect, that can reduce the concentration of the saliva-introduced bacteria in the sampled blood below the detectable threshold. Therefore, a delay in detection of *Bartonella* in the blood used for tick feeding may be due to (a) a necessary reactivation period, (b) a 48-hour delay in initiation of actual tick feeding, or (c) a gradual increase of the number of attached feeding ticks and consequently of the volume of infected tick saliva injected into the feeder.

In addition, there is the possibility of proliferation of the saliva-introduced agent in the blood contained within the membrane feeder. However, because the blood in the feeder was completely replaced every day, if some bacteria were inoculated within the first 48 hours after placement of ticks on a membrane, they would have the same chance for growth and detection as those inoculated and detected each day after 72 h hours.

The molting success of larvae fed upon a *Bartonella*-infected mouse was low, and molted nymphs were not tested due to their paucity. Therefore, the prevalence of infection in molted ticks and the efficiency of transstadial transmission could not be accessed directly. Nevertheless, each of the three mice exposed to nymphs from the infected cohort became infected with Bartonella, even those on which only two and three ticks successfully fed to repletion. This suggests that the prevalence of infection in this cohort of nymphs was 40% or higher.

On the other hand, all four of the partially engorged female ticks examined at 72 hours after placement on a membrane feeder contained bacteria in the muscle and salivary gland tissues, but not in the midgut. These results imply a passage of *B. birtlesii*, acquired with the blood meal, through the epithelial cells of the gut during or after the acquisition-feeding followed by dispersal of bacteria throughout the body of the tick including the muscle cells. It also indicates that each of the females was infected during the nymphal feeding and retained the infection through both the molt and the following three-month long period of starvation. Therefore, it appears that the efficiency of both the acquisition of *B. birtlesii* by *I. ricinus* larvae and nymphs from an infected host, and of the transstadial transmission is high.

Absence of *B. birtlesii* in the midgut of the tick is in contrast with the known distribution of other bartonellae in Anoplura and Siphonaptera vectors. For example, *B. quintana* inhabits the louse intestinal lumen and is excreted in louse feces throughout the lifespan of an infected human body louse [30]; and *B. henselae* remains in the gut of the cat flea – *C. felis* for up to 9 days [31]. The lack of *B. birtlesii* in the midgut of feeding ticks and its presence in the salivary glands confirms that its transmission to the host occurs with saliva and not through contaminated feces. It remains to be studied whether initiation of the next feeding is necessary for bacterial invasion of salivary glands and the subsequent transmission into a susceptible host.

Together, results of this study demonstrate that both larval and nymphal *I. ricinus* are capable of acquiring *B. birtlesii* from an infected host, transmitting it through the molt to the next life stage, maintaining the infection for several months of starvation, and ejecting it with saliva during the subsequent feeding. Using a murine model, we show for the first time the ability of the erythrocyte-associated bacterium to survive and disseminate in a tick vector, where it escapes from the midgut into the hemocoel and infects salivary and muscle tissues.

This work represents the first *in vivo* demonstration of a *Bartonella* sp. transmission by ticks. It does not claim that ticks are principal vectors of *Bartonella* spp, but it does corroborate a prospect that ticks play a role in the natural cycles of some of the bartonellae including those pathogenic for humans. Consequently, bartonelloses should be included in the differential diagnosis for patients exposed to tick bites.
